# Estimated Costs of 4-Month Pulmonary Tuberculosis Treatment Regimen, United States

**DOI:** 10.3201/eid2910.230314

**Published:** 2023-10

**Authors:** Carla A. Winston, Suzanne M. Marks, Wendy Carr

**Affiliations:** Centers for Disease Control and Prevention, Atlanta, Georgia, USA

**Keywords:** tuberculosis and other mycobacteria, bacteria, bacterial infections, treatment regimen, cost assessment, United States

## Abstract

We estimated direct costs of a 4-month or 6-month regimen for drug-susceptible pulmonary tuberculosis treatment in the United States. Costs were $23,000 per person treated. Actual treatment costs will vary depending on examination and medication charges, as well as expenses associated with directly observed therapy.

In 2022, the Centers for Disease Control and Prevention (CDC) published recommendations for a 4-month tuberculosis (TB) treatment option using rifapentine and moxifloxacin for persons >12 years of age with drug-susceptible pulmonary TB (*1*,*2*). By using published data sources for US healthcare costs for TB, we estimated costs per person treated with the 4-month treatment regimen and a standard 6-month regimen (*3*).

TB treatment consists of an intensive phase for bactericidal and sterilizing activity, followed by a continuation phase to ensure sterilization. Compared with a standard 6-month regimen, the 4-month regimen replaces rifampin and ethambutol in the 8-week daily intensive phase with high-dose rifapentine and moxifloxacin. The 4-month regimen continuation phase of 9 weeks of daily rifapentine, isoniazid, and moxifloxacin compares with a continuation phase of 18 weeks of daily rifampin and isoniazid for the 6-month regimen. For both regimens, recommendations are to administer treatment under directly observed therapy (DOT) for 5 of 7 weekly doses throughout treatment (*1*,*3*).

## The Study

We estimated direct healthcare costs to treat 1 person with drug-susceptible TB (Table). Direct healthcare costs include inpatient and outpatient costs associated with diagnosis and treatment and exclude societal costs such as the value of lost patient productivity. All costs for this analysis were based on published unit cost data and updated to 2021 dollars by using Bureau of Economic Analysis Personal Consumption Expenditures indices for hospital and outpatient services (*4*). Overall, TB outcomes and adverse events were similar in the clinical trial of the 4-month regimen compared with the 6-month regimen (*2*); therefore, we assumed the percentage of persons hospitalized at any point during TB diagnosis and treatment was equivalent across regimens. We assumed hospitalization costs of persons with TB on both regimens to be equivalent at $17,432 in 2021 dollars, reflecting an estimated 49% of persons with TB hospitalized at $1,482 per day for an average 24 days and including 20% for physician costs, on the basis of previous publications (*5*–*7*). We added outpatient TB disease costs, including examination costs, clinic supplies, medication costs, and DOT costs, to drug-susceptible TB inpatient costs. We used medication costs based on US Department of Veterans Affairs acquisition costs (*8*) for maximum adult doses (isoniazid 300 mg, $0.19/dose; rifampin 600 mg, $2.45/dose; rifapentine 1,200 mg, $14.44/dose; pyrazinamide 2,000 mg, $17.88/dose; ethambutol 1,600 mg, $1.13/dose; moxifloxacin 400 mg, $2.35/dose) and applied them to 7-day-per-week dosing. DOT costs assumed clinical follow-up and personnel costs at $24/visit updated to $28/visit in 2021 dollars, on the basis of in-person observation 5 days per week (*6*).

**Table:** Input characteristics and direct treatment costs estimated for pulmonary drug-susceptible TB treatment, United States*

Characteristic	4-month regimen			6-month regimen	
Intensive phase	Continuation phase	Intensive phase	Continuation phase
Time (doses)	8 wks (56 doses)	9 wks (63 doses)		8 wks (56 doses)	18 wks (126 doses)
Anti-TB medications	Isoniazid 300 mg, rifapentine 1,200 mg, pyrazinamide 2,000 mg, moxifloxacin 400 mg	Isoniazid 300 mg, rifapentine 1,200 mg, moxifloxacin 400 mg		Isoniazid 300 mg, rifampin 600 mg, pyrazinamide 2,000 mg, ethambutol 1,600 mg	Isoniazid 300 mg, rifampin 600 mg
No. daily pills†	15	11		12	4
No. DOT clinic visits‡	40	45		40	90
Costs§					
Examination	370			402	
Clinic supply	74			80	
Medication	3,023			1,546	
DOT	2,354			3,600	
Subtotal outpatient costs	5,820			5,628	
Subtotal inpatient costs¶	17,432			17,432	
Total estimated direct treatment costs	23,252			23,060	

*DOT, directly observed therapy; TB, tuberculosis. †Pill count assumed anti-TB medications plus 1 daily pyridoxine (vitamin B6) tablet for persons taking isoniazid. ‡DOT administered 5 of 7 weekly doses throughout treatment. §In 2021 US dollars. ¶Inpatient costs assumed equivalent across regimens because TB outcomes and adverse events were similar according to clinical trial data.

We estimated examination costs by using Centers for Medicare and Medicaid Services average allowable charges for laboratory (*9*) and physician services (*10*). Baseline examination costs before initiating therapy, regardless of regimen, assumed 3 sputum smears for acid-fast bacilli at $5.39 per smear and 3 cultures for *Mycobacterium tuberculosis* at $10.80 per culture, 1 phenotypic drug-susceptibility test panel to include at least each drug in the regimen at $7.31 per drug, 1 rapid molecular drug-resistance detection panel to include at least each drug in the regimen at $41.68 per drug, 1 two-view chest radiograph at $34.20, 1 complete blood count at $7.77, 1 comprehensive metabolic panel at $10.56, and 1 liver function test at $8.17. We assumed smears and cultures were repeated monthly throughout treatment and yielded $16/month in additional laboratory costs.

## Conclusions

In the absence of data from programmatic use of the 4-month regimen, estimated total direct costs of the 4-month and 6-month regimens in the United States based on published sources were similar, at $23,000 per person treated for pulmonary drug-susceptible TB. Hospitalization during diagnosis and treatment accounted for three-quarters of total costs. Because we assumed that hospitalization proportion was equal for the 4-month and 6-month regimens on the basis of adverse events reported during the clinical trial (*2*), inpatient costs were equal between regimens. Cost differences between the regimens were driven by higher drug costs in the 4-month regimen and by higher outpatient costs for length of follow-up and DOT in the 6-month regimen. In terms of drugs, rifapentine costs more per dose than rifampin, but fewer total doses are required for the 4-month regimen. Lower rifapentine prices would decrease costs associated with the 4-month regimen. The longer treatment regimen results in additional clinical follow-up assumptions, including 2 additional smears and 2 additional cultures, and greater DOT costs compared with the shorter regimen. However, some TB programs limit DOT in the continuation phase (e.g., 3 times/week) of the 6-month regimen, or may opt to use video DOT, which costs less than in-person DOT, for either the 4-month or 6-month regimen (*3*,*11*,*12*). Although we estimated molecular drug-susceptibility testing for moxifloxacin in this analysis as having equivalent cost to other drug-susceptibility tests, it might not yet be widely available in first-line testing because fluoroquinolones have historically been used primarily for drug-resistant TB treatment (*3*). Susceptibility testing for moxifloxacin as a first-line drug could result in start-up costs, changes in workflow, or a need for clinical validation if tests are not already in use by or accessible to local programs. CDC’s Molecular Detection of Drug Resistance service offers sequencing at no charge to US public health laboratories to enhance early detection of mutations (*13*).

Societal costs attributable to lost patient productivity are likely to be higher for the 6-month versus the 4-month regimen as a function of longer healthcare engagement. Because this analysis focused on healthcare system costs, we did not include patient or larger societal costs such as costs associated with premature death or sequelae from TB. Adverse event reporting and death while receiving treatment during the clinical trial was statistically equivalent between the 4-month and 6-month regimens, with no signals of higher likelihood of relapse with the shorter regimen (*2*). On the basis of the trial data, we therefore could expect societal costs from premature death to be similar across regimens, thus providing no differentiation between regimens; however, post-TB disease or death was not assessed. Prior analyses, updated to 2021 dollars, assumed $29.55 for patient time for travel to and from the clinic and $3.64/dose for doses administered under in-person DOT and an additional $2.45 value per daily dose of patient time in physically ingesting medication (*6*). Including patient costs for in-person DOT would yield ≈$1,500 ($1,456) greater costs for the 6-month regimen compared with the shorter regimen. Video DOT might mitigate patient costs (*11*,*12*).

Treatment shortening that has similar success and safety in producing favorable TB outcomes has potential advantages from the healthcare perspective and may be appealing to persons with TB if it reduces healthcare engagement. Actual treatment costs will vary across different settings (*14*) depending on examination and medication charges and the method of DOT for treatment support. Because of the limits of the data available to us, which are published list prices for unit costs, we were unable to estimate uncertainty around cost estimates for components of TB care. We encourage TB programs and researchers to report primary data collection on healthcare system costs and costs experienced by persons with TB to provide better data for costs for the new 4-month regimen in diverse real-world settings.
